# Relational aggression in romantic relationship: empirical evidence among young female adults in Malaysia

**DOI:** 10.1186/s40359-024-01670-4

**Published:** 2024-05-28

**Authors:** Mohammad Rahim Kamaluddin, Shalini Munusamy, Chong Sheau Tsuey, Hilwa Abdullah & Mohd Nor

**Affiliations:** 1 Department of Psychology, School of Medicine, International Medical University, Bandar Bukit Jalil, 57000 Kuala Lumpur, Malaysia; 2 Centre for Research in Psychology and Human Well Being, Faculty of Social Sciences and Humanities, National University of Malaysia, 43600 Bangi, Selangor Malaysia

**Keywords:** Relational aggression, Romantic relationship, Social support, Personality trait, Loneliness, Attachment style

## Abstract

**Background:**

Aggressive behaviour in romantic relationship is a social problem of great concern. Studies related to the influence of psychosocial factors on relational aggression are still limited. Furthermore, these factors have not been widely studied in the local context, resulting in the issue of relational aggression among young female adults still not being addressed. This study aims to explore whether psychosocial factors such as big five personality traits, adult attachment style and loneliness could predict relational aggression in romantic relationships among young female adults in Malaysia. In addition, this study aims to identify the moderating effect of social support in the relationship between psychosocial factors and relational aggression in romantic relationship.

**Methods:**

A quantitative research approach was used with 424 young female adults in Malaysia aged between 18 and 30 years old (mean age = 24.18) were recruited through multistage sampling design by completing a questionnaire consisting of the Big Five Inventory (BFI), Experiences in Close Relationships Scale II (ECRS-II), Revised UCLA Loneliness Scale, Measure of Relational Aggression and Victimization (MRAV) and Multidimensional Scale of Perceived Social Support (MSPSS).

**Results:**

Multiple regression analysis predicted significant relationship between agreeableness personality, loneliness, avoidant attachment style and anxious attachment style with relational aggression in romantic relationships. Hierarchical regression analysis found a significant effect of social support as a moderator between loneliness with relational aggression in romantic relationships.

**Conclusions:**

Thus, the results show that young female adults with low level of agreeableness, high level of loneliness, avoidant attachment style and anxious attachment style are at a higher risk of engaging in relational aggression in romantic relationships. The implication of this study can help in understanding the psychosocial factors that form the basis of relational aggression in romantic relationships. Hence, the gap in knowledge warrants further research.

## Background

The development of romantic relationships among early adulthood is crucial in forming views about intimate relationships and exhibiting intimacy, power, and control [1]. Emerging adulthood is a key developmental stage for creating a healthy romantic relationship. Some romantic relationships involve aggressive behaviour between partners, which can manifest in various forms such as physical, non-physical, direct, or indirect aggression, overt or covert aggression [2]. Aggressive behaviour is a criminogenic trait linked to various violent crimes including dating violence [3]. Physical aggression involves intentionally using physical force to hurt the partner, ranging from mild actions like pushing to severe violence like choking, slapping or weapon use [4]. Emotional abuse is also a common form of abuse in romantic relationships [5]. The online dating scam is another alarming form of dating violence that can result in financial loss and severe emotional and psychological suffering (6–7). Relational aggression is a form of non-physical and covert aggression, involves threatening others by manipulating and acting to jeopardize romantic relationships [8]. Unlike physical aggression, relational aggression occurs without any physical force or physically threatening the individual and can be considered a type of psychological aggression, targeting perceptions, feelings, or behaviour in romantic relationship [9]. Relational aggression can be indirect, such as through negative facial expressions or spreading rumors about a partner. While there has been extensive research on physical aggression and violence in romantic relationships [10–12], there is relatively less research on relational aggression in romantic relationships.

Relational aggression in romantic relationships might appear as threats to end the relationship if the other person doesn’t cooperate, flirting with other people to make the other person envious, or treating the other person silently while upset [9]. In terms of relational aggression, females who utilized high levels of relational aggression had a strong tendency to see other people’s acts as hostile and malevolent, whereas males did not [13]. Examining relational aggression and its relationship with adaptive functioning in females may shed light on the critical mechanisms involved in females’ dating violence. In this study, we hope to study the psychosocial factors most related with relational aggression in females by looking at components known to relate to aggression in females, such as individual characteristics and environmental factors. There is little evidence from research on female gender to differentiate the experience of relational aggression in romantic relationships, female perpetrators will be the greatest risk of this aggressive behaviour and young female adults may experience greater psychological stress than men (13–14). Therefore, this study focuses only on female samples and will be done using Malaysian samples. Despite research, little is known about how relational aggression originate, persist, and have an impact on romantic relationships, including whether men and women experience these issues differently (13–14). Romantic relational aggression has also been linked to relationship quality, violence, psychosocial maladjustment, impulsivity, hostile attribution biases, loneliness, emotional sensitivity to relational incitements, and abuse history [13].

In addition, this study emphasizes the psychosocial aspect of a person that can cause the tendency to behave aggressively in romantic relationships. It is important to identify the psychosocial aspects of a person who tends to engage in relational aggression in romantic relationships. The link between relational aggression and psychosocial factors such as loneliness, attachment styles, and personality type has been established (15–16). Personality traits of aggressors have been known to be associated with dating violence (15–16). This study used the “Big Five” personality model (extraversion, agreeableness, openness, conscientiousness, and neuroticism) as one of the psychosocial factors. Each main trait from this model can be divided into several aspects to provide a more detailed analysis of a person’s personality. Several theorists argue that personality variable is an important predictor of aggressive behaviour in romantic relationship [17–19]. Agreeableness dimensions are often associated with aggressive behaviour [18, 20, 21]. Besides that, a study conducted by Ulloa et al. (2016) found individuals with a high neuroticism personality tend to be victims in relational aggression during intimate relationships [22]. The findings of this study are also supported by other research that neuroticism trait as the main personality trait that gives a strong influence on relational aggression (23–24).

In addition to personality traits, other factors such as the level of loneliness are also considered to be a strong predictive factor of relational aggression especially the tendency to be a victim [25]. Generally, loneliness can be associated with individuals having a lack of social support as well as showing no interest in social networks [25]. Many studies have linked aggressive behaviour with loneliness [26–28]. Loneliness is defined as a negative emotional response to the discrepancy between the desired and achieved quality of one’s social network [27]. In addition, relational aggression is caused by the loneliness faced by an individual [28]. Individuals who are lonely describe themselves negatively and have negative ideas about others. As a result, loneliness leads to a bad perception of oneself, such as being unwanted and unaccepted by others, and it leads to aggression, which is a means of using force to influence other people in interpersonal relationships [29]. Individuals with high level of loneliness are at high risk of engaging in relational aggression in romantic relationship (30–31). Another psychosocial aspect often associated with relational aggression is attachment style. Attachment style is said to be able to shape the probability of an individual being involved in incidents of relational aggression in romantic relationship.

An expanding corpus of research has highlighted attachment theory as a crucial paradigm for comprehending emotional and interpersonal processes that take place across the lifespan [32–34]. The foundation of attachment theory is the idea of an attachment behavioural system, in which attachment actions are grouped together to strengthen a particular attachment figure. A sense of personal security within the relationship can be established or maintained by intimate partner violence, according to the attachment theory. People feel startled when they sense a threat to their attachment connection, and the ensuing anxiety causes them to act in ways that protect their attachment system [35]. Individuals with different attachment style also have an influence strongly to the involvement of individuals in the occurrence of aggression (36–37). Besides that, individuals with avoidant attachment shows high relational aggression in romantic relationship (38–39). Besides that, individuals who often exhibit anxious attachment to their partners such as fear of rejection and dependency on their partner are more likely to experience relational aggression in romantic relationships (40–41).

## The potentially moderating role of Social Support

In relation to that, social support is used as a moderator based on previous literature studies [42–44]. Social support is also defined as interpersonal relationships and support provided by social groups that aim to provide well-being to individuals [42]. Social support from family and friends is important in contributing to positive psychological health among early adulthood and influences the act of aggressive behaviour [45]. Previous studies have shown that social support has a significant relationship with big personality traits, especially with extraversion and agreeableness [45–49]. In addition, a few studies also found that family members with agreeableness trait also provide more social support [46–48]. Besides that, people who experience loneliness interact less with friends and family than people who do not feel lonely. In other words, the less social support a person has, the higher the level of loneliness [50]. According to earlier research, there have been negative association between relational aggression and social support as well positive association between relational aggression and psychosocial maladjustment during major developmental stages including childhood, adolescence, and young adulthood [51–53].

According to research, individuals with little social support from their parents were more likely to engage in verbal, physical, and relational aggression [54] whereas individuals who reported high perceived social support from peers were less likely to engage in overt and relational aggression [55]. Besides that, individuals who have supporting friends and family have lower relational aggression. Family and peer support can help to mitigate the harmful effects from using relational aggression behaviour in their romantic relationship. Adults with high levels of social support outperformed those with low levels of family and peer support in exhibiting relational aggression behaviour in romantic relationships [56]. Although both relational aggression and social support are empirically connected to maladjustment, research on the interaction effect of psychosocial factors and social support on relational aggression is still limited (57–58).

Besides that, a study done in US had found that there is no evidence of social support act as a moderator between psychosocial factors and dating violence [59]. Only a small amount is allocated in the extent literature to research the triad of the relationship. In accordance with that, this study will further explore to develop an understanding of the role of social support in the association between psychosocial factors and relational aggression. Among several theories of social behaviour, for this study we have used Albert Bandura (1986) social cognitive theory to help provide researchers with a comprehensive framework to understand the factors that may influence aggressive human behaviour. Although Bowlby (1969) prioritized and focused on understanding the nature of caregiver’s relationship with his infant, at the same time he also believed that bonding features are present in human life experience from “cradle to grave” [30]. Besides that, attachment style and social support combine the theory-based prediction that people with an insecure attachment style are more likely to evaluate others’ reactions negatively [60].

This study can give awareness to young female adults about the issue of relational aggression that can happen in a romantic relationship. This is because relational aggression is an issue that is not given attention in romantic relationships by women and only aggressive behaviour such as physical and sexual is considered more harmful in romantic relationships. This study can give awareness to young female adults about the characteristics of an individual who practices relational aggression in a romantic relationship and can help in finding a solution from practicing relational aggression in romantic relationship. This study can also help young adults to identify this issue so that it does not continue and affect romantic relationships in adulthood. Relational aggression is known to be a relevant social problem factor which can be a precursor to abusive romantic relationships in later adulthood [61].

A conceptual framework in this study was built based on the social cognitive theory introduced by Albert Bandura in 1986, attachment theory developed by John Bowlby (1907–1990) and the big five personality theory developed in 1949 by D. W. Fiske (1949) as well as from the findings of research on previous studies in the field of psychosocial factors and relational aggression in romantic relationship. In general, this study aims to explore whether psychosocial factors could predict relational aggression in romantic relationships. There is not much direct research that examines covert set of manipulative behaviors in romantic relationships such as relational aggression. Besides that, there are only a few studies conducted in Malaysia about relational aggression in romantic relationships compared to studies conducted in Western countries [53–55, 60–62]. Therefore, it is important to conduct this study using respondents from Malaysia so that it can help psychologists and other parties involved to identify individuals using relational aggression in romantic relationships and from being involved in psychological problems.

## The present study

This study was designed to explore whether psychosocial factors such as big five personality traits, attachment style and loneliness could predict relational aggression in romantic relationship among young female adults in Malaysian context and aims to extend findings from previous studies in this field. The researchers hypothesize that psychosocial factors, such as personality trait, attachment styles, and loneliness, will play a significant role in determining the presence and severity of relational aggression in romantic relationships. In addition, it is believed that social support will act as a moderating factor in the relationship between psychosocial factors and relational aggression. As a result, this study aims to shed light on the drivers behind relational aggression in romantic relationships and to better understand the relationship between psychosocial factors and relational aggression. This study is regarded novel because there are no known studies on relational aggression in romantic relationship in the Malaysian context as this will be the first Malaysian study to define the relational aggression in romantic relationship among the sample of young female adults in Malaysia.

## Methods

### Participants

An online survey was conducted with a total of 424 females from early adulthood stage, aged between 18 and 30 years old in Malaysia. According to DOSM (2021), the total population of women in early adulthood in Malaysia is 15,758.2(‘000). From the entire population in each state, the respondents aged between 18 and 30 were selected in this study using Raosoft formula. Proportionate stratified random sampling was used to recruit respondents from 13 states in Malaysia to get sufficient sample size from each state through Raosoft formula calculation in July 2022. Then, convenience sampling was used to select a study sample from the population to get a sufficient sample from each state where an advertisement was posted in social media. Inclusion criteria: [1] participants must be Malaysian; [2] female participants aged between 18 to 30 years old only; [3] currently in a romantic relationship for more than 3 months; [4] must answer all questions in relation to the most recent partner or romantic relationship; [5] informed and voluntary participation in the study. The study sample for this research consists of different races, occupation, and education background so that they will have equal opportunity to be selected as a respondent.

### Instruments

#### Big five inventory (BFI)

The Malay version of Big Five Inventory (BFI; 63) which was developed by Muhammad et al., [63] was used to measure the five basic personality dimensions, namely extraversion, agreeableness, conscientiousness, openness, and neuroticism. The 44-item BFI is rated on a 5-point Likert Scale from 1 (strongly disagree) to 5 (strongly agree). After reverse scoring, the mean score of each subscale is obtained. The Malay version of the BFI shows good internal consistency, convergent and discriminant validity [63]. The internal reliability of this scale in the current study was high, with a Cronbach’s alpha calculation of 0.78 to 0.88 with a mean of 0.81.

#### UCLA loneliness scale-3

The Malay version of the Rusell’s [64] UCLA Loneliness Scale [65] was used to measure loneliness. This tool consists of 20 items and is rated on a 4-point Likert scale from 1 (Never) to 4 (Always). Loneliness was assessed by averaging the scores of all items with higher scores indicating higher levels of loneliness. The internal reliability of this scale in the current study reported with a Cronbach’s alpha of 0.83.

#### Experiences in close relationships– II (ECR-II)

The Experiences in Close Relationships Scale II (ECRS-II; 67) assessed individual differences in anxious attachment style (i.e., the extent to which individuals feel secure versus insecure about romantic partner relationships and reactions) and avoidant attachment style (i.e., the extent to which individuals feel uncomfortable with having close relationships with others versus feel safe to rely on others). The Malay version of the ECR-II [66] was used in this study. The internal reliability of this scale in the current study was high, with a Cronbach’s alpha calculation of 0.82 to 0.83 with a mean of 0.83.

#### Measure of relational aggression and victimization (MRAV)

This instrument was developed by Linder et al. [67]. This 56-item instrument consists of six subscales that measure six dimensions of aggression, namely relational aggression, physical aggression, relational victimization, physical sacrifice, exclusivity, and prosocial behaviour. For this study, only the subscales of relational aggression (5 items) were used. Items in this tool are rated on a 7-point Likert-type scale from 1 (Not at all True) to 7 (Very True). This questionnaire was translated into Malay language using Forward-Backward translation method and followed by content validation. CVR technique was used to measure the content validity of this questionnaire. The CVR was in the range 0.7-1 for all items and the overall mean CVR values were 0.83. According to Rahim et al. [68], in the context of measuring psychological test, tools which are available in their own native language will be more appropriate and measurement will be more accurate compared to other languages. The internal reliability of this scale in the current study was high with a Cronbach’s alpha calculation of 0.88 with a mean of 0.89.

#### Multidimensional scale of perceived social support (MSPSS)

This questionnaire was developed by Zimet et al. [69] and was used to measure social support of an individual. The MSPSS consists of 12 items assessing three specific sources of social support namely family, friends, and others. This test tool uses a 7-point Likert scale where (1 = strongly disagree, 7 = strongly agree). In this study, the Malay version of the MSPSS tool was used which was translated and validated by Ng et al., [70]. The internal reliability of this scale in the current study was high, with a Cronbach’s alpha calculation of 0.93.

### Procedure

The survey was conducted from July 1 to July 26, 2022. According to Connelly [71], previous studies suggest that the sample size of the pilot study should be 10% of the sample size used for the actual study. Therefore, a pilot study was carried out before the real study with 44 respondents in the state of Selangor. The researcher chose Selangor because it is the state where the researcher is currently living, and this will make it easier to carry out the study. In the actual study, 424 participants were recruited based on Table 2. This study was approved by the Research Ethics Committee of The National University of Malaysia (No: 2022 − 549). All participants were informed of the research objectives and their rights on the first screen (voluntary participation, the right to withdraw at any time and anonymity). This study was not conducted with any minors. At the start of the test, informed permission was acquired, this study only moved forward if the subject ticked the box that said, “Yes, I offer my consent to participate.” The participants’ privacy was guaranteed by the test’s anonymity and the numerical coding of their replies.

### Data analysis

Descriptive statistics and inferential statistics were calculated using SPSS 26.0. For inferential statistics multiple regression and hierarchical regression has been used in this study. Multiple regression was used to explore whether psychosocial factors such as big five personality traits, attachment style and loneliness could predict relational aggression in romantic relationship. A single dependent variable and numerous independent variables can be analysed using the statistical method known as multiple regression. The value of R, the multiple correlation coefficient, is shown in the “R” column. The “R Square” column displays the R^2^ value, also known as the coefficient of determination, which is the percentage of the dependent variable’s variance that can be explained by the independent variables. R can be thought of as one indicator of the accuracy of the dependent variable’s prediction [72]. It is the proportion of variation accounted for by the regression model above and beyond the mean model. Hierarchical regression was used to study the effect of social support as a moderator in the relationship between psychosocial factors (personality trait, attachment style and loneliness) with relational aggression in romantic relationship. The moderation effect analysis was carried out using SPSS hierarchical regression. The hierarchical regression is a more appropriate method for determining whether a quantitative variable has a moderating effect on the relationship between two other quantitative variables [72]. If the moderation test result fell within the 95% confidence interval and contained 0, it meant that the moderation impact of social support was not significant; if it did not, it meant that the moderation effect of social support was substantial. In this study, *p* <.05 was regarded as statistically significant. In this study, SPSS 26.0 software were used to analyse the data.

## Results

### Descriptive statistics

A total of 500 participants have completed the online survey but only 424 (M ± SD = 24.18 ± 3.21 years) participants’ responses were included after 76 questionnaires were rejected from this study as it did not meet the inclusion criteria. The highest level of education obtained by the participants is degree education. 18.2% of participants had engaged in aggression towards their romantic partner.

**Table 1:** Demographic information of the participants

Profile	Distribution (n)	Percentage (%)
Age		
18–22	149	36
23–27	201	47
28–30	74	17
**Level of Education**		
High School	19	4.5
Diploma/Foundation/STPM	41	9.7
Degree	197	46.5
Masters	152	35.8
PhD	15	3.5
**Satisfaction in Romantic Relationships**		
Yes	353	83.3
No	71	16.7
**Experiencing Aggression from partner**		
Yes	64	15.1
No	360	84.9
**Showing Aggression towards partner**		
Yes	77	18.2
No	347	81.8
**Have you ever been physically abused by your partner**		
Yes	17	4.0
No	407	96.0
**Have you ever been sexually abused by your partner**		
Yes	13	3.1
No	411	96.9

### Inferential statistics

Table 2 shows the results of a multiple regression analysis in predicting relational aggression based on big five personality traits, attachment styles, and loneliness among young female adults in Malaysia. Among the five subscales of personality trait, agreeableness showed a significant predictor. In addition, loneliness, avoidant attachment style, and anxious-attachment style also showed significant prediction with relational aggression. Overall, the results of the regression analysis showed that agreeableness, loneliness, avoidant attachment style, and anxious attachment style together can predict 30.3% of the variance in relational aggression (R²=0.303), where [F (3,269) = 22.561, *p* < 0 0.05]. The subscale of agreeableness showed negative prediction (β=-0.305, *p* <.05) with relational aggression whereas loneliness (β = 0.364, *p* <.05), avoidant attachment style (β = 0.420, *p* <.05), and anxious attachment style (β = 0.321, *p* <.05) showed positive prediction with relational aggression. These findings showed that higher level of agreeableness trait contributes to lower level of relational aggression in romantic relationships. Besides that, high levels of loneliness, avoidant attachment style, and anxious attachment style contribute to higher level of relational aggression in romantic relationship.

For hierarchical regression analysis, only those variables that were significant in the multiple regression analyses were entered into hierarchical regression models which are agreeableness trait, loneliness, avoidant attachment style, and anxious attachment style. Table 3 shows the hierarchical regression analysis where R² value for Model 1 is 0.097, F (25.735) = 22.545, *p* <.05. This means that the agreeableness dimension accounts for 9.7% of the variance in relational aggression. While the R² value obtained for Model 2 is 0.098, F (17.410) = 15.240, *p* <.05. This means that social support and agreeableness dimensions contribute as much as 9.8% of the variance to relational aggression in romantic relationships. These results showed that the percentage of variance only increases by 0.1% (9.8%– 9.7%) with the presence of a moderator in this model. The results in Table 3 showed that the dimension of agreeableness as a predictor is significant with a value of β =-0.296, t = -6.333, *p* <.05. While social support as a predictor is not significant with β value = -0.062, t = -1.331, *p* >.05. After entering the moderator, the interaction term of social support and agreeableness is not significant with a value of β = -0.406, t = − 0.816 and *p* >.05. The agreeableness subscale was a significant predictor in the first block (*p* <.05) but did not reach significance in the second block (*p* =.415).

Table 4 shows the hierarchical regression analysis where R² value for Model 1 is 0.135, F (35.826) = 32.761, *p* <.05. This means that the loneliness level dimension accounts for 13.5% of the variance in relational aggression. While the R² value obtained for Model 2 is 0.146, F (25.874) = 23.915, *p* <.05. This means that social support and loneliness level dimensions contribute as much as 14.6% of the variance to relational aggression in romantic relationships. These results show that the percentage of variance only increases by 1.1% (14.6%– 13.5%) with the presence of a moderator in this model. The results in Table 4 show that the dimension of loneliness as a predictor is significant with a value of β = 0.383, t = 7.767, *p* <.05. While social support as a predictor is not significant with a value of β = 0.048, t = 0.964, *p* >.05. After entering the moderator, the interaction term of social support and loneliness is significant with a value of β = 0.550, t = 2.349 and *p* <.05. The loneliness subscale was a significant predictor in all blocks (*p* <.05), with *p* =.019 in the second block.

Table 5 shows the hierarchical regression analysis where R² value for Model 1 is 0.231, F (40.936) = 42.014, *p* <.05. This means that the attachment style dimension accounts for 23.1% of the variance in relational aggression. While the R² value obtained for Model 2 is 0.237, F (25.225) = 25.976, *p* <.05. This means that social support and attachment style dimensions account for 23.7% of the variance in relational aggression in romantic relationships. These results show that the percentage of variance only increases by 0.6% (23.7%– 23.1%) with the presence of a moderator in this model. The results in Table 5 show that the dimension of avoidant attachment style as a predictor is significant with a value of β = 0.368, t = 8.345, *p* <.05 and the dimension of anxious attachment style as a predictor is significant with a value of β = 0.244, t = 5.364, *p* <.05. While social support as a predictor is.

not significant with a value of β = 0.20, t = 0.447, *p* >.05. After entering the moderator, the interaction term of social support and attachment style was not significant on the relational aggression with values ​​of β = 0.155, t = 0.676, *p* >.05 and β = 0.520, t = 2.925, *p* >.05. The ECR’s anxious and avoidant subscale were significant predictor in the first block (*p* <.05) but did not reach significance in the second block (*p* =.328;0.105).

**Table 2:** Multiple Regression

Variables	B	SE b	β	t	Sig
Extraversion	0.031	0.065	0.024	0.470	0.639
Agreeableness	− 0.292	0.089	− 0.170	-3.282*	0.001
Conscientiousness	− 0.104	0.073	− 0.080	-1.430	0.153
Neuroticism	− 0.069	0.068	− 0.054	-1.018	0.309
Openness	0.121	0.072	0.074	1.674	0.095
Loneliness	0.103	0.033	0.156	3.142*	0.002
Avoidant-attachment style	0.095	0.014	0.311	6.806*	0.000
Anxious-attachment style	0.058	0.015	0.168	3.824*	0.000

*p <.05

**Table 3:** Hierarchical Regression

Model 1							
(Constant)	5.653	0.489		11.570	0.311	0.097	0.092
Agreeableness	− 0.916	0.145	− 0.296	-6.333*			
Social Support	− 0.068	0.051	− 0.062	-1.331			
**Model 2**							
(Constant)	3.931	2.165		1.816	0.313	0.098	0.092
Agreeableness	− 0.377	0.675	− 0.122	− 0.559			
Social support	0.306	0.462	0.279	0.663			
Agreeableness X Social support	− 0.117	0.143	− 0.406	− 0.816			

*p <.05

**Table 4:** Hierarchical Regression

Model 1							
(Constant)	0.121	0.437		0.276	0.367	0.135	0.131
Loneliness	1.009	0.130	0.383	7.767*			
Social support	0.052	0.054	0.048	0.964			
**Model 2**							
(Constant)	2.757	1.203		2.291	0.382	0.146	0.140
Loneliness	− 0.244	0.548	− 0.093	− 0.444*			
Social support	− 0.501	0.242	− 0.456	-2.074			
Loneliness X Social support	0.268	0.114	0.550	2.349*			

*p <.05

**Table 5:** Hierarchical Regression

Model 1							
(Constant)	0.248	0.340		0.730	.480a	0.231	0.225
Avoidant-attachment style	0.405	0.048	0.368	8.345*			
Anxious-attachment style	0.301	0.056	0.244	5.364*			
Social support	0.022	0.049	0.020	0.447			
**Model 2**							
(Constant)	0.111	1.068		0.104	.487b	0.237	0.228
Avoidant-attachment style	0.170	0.251	0.155	0.676			
Anxious-attachment style	0.642	0.220	0.520	2.925			
Social support	0.054	0.210	0.049	0.255			
Avoidant-attachment style X Social support	0.049	0.050	0.251	0.979			
Anxious-attachment style X Social support	− 0.074	0.045	− 0.296	-1.622			

*p <.05

## Discussion

The participants that have been selected for this study are young female adults between the age of 18 to 30 (M ± SD = 22.08 ± 3.21 years) who are currently in a romantic relationship for more than three months. Regression analysis was done, and it was found that only agreeableness trait showed significant predictor on relational aggression in romantic relationship and the other four dimensions of the big five personality in the psychosocial factor variable, which are extraversion, openness, neuroticism, and conscientiousness are not predictors or contributors to relational aggression in romantic relationships. Therefore, the findings prove that the importance of the relative contribution of personality traits of agreeableness. Generally, in an interpersonal context, personality is known to play an important role in determining the likelihood of engaging in an aggressive act. Negative emotions are generally harmful to romantic relationships. The result from our study is contradictory with the research findings by Burton et al. [73] where they have found that higher relational aggression was associated with higher levels of neuroticism and lower level of conscientiousness.

In addition, in some studies it has been found that individuals who tend to engage in relational aggression are more likely to show lower traits of agreeableness, openness and conscientiousness [66–77]. In our study, none of the big five personality traits except for agreeableness show significant prediction towards relational aggression in romantic relationships. This may be due to in general agreeableness traits may have stronger predictive utility than other personality traits (78–79). It has also been shown that agreeableness trait is negatively associated with relational aggression [80–82]. Agreeableness characterized as cooperation and understanding is an aspect related to motivation to maintain positive interpersonal relationships [83]. Likewise, the relationship between agreeableness and mind suggests that the former is responsible for processing social information.

Furthermore, agreeableness supports altruism while relational aggression is a type of destructive and hostile behaviour that has anti-social tendencies [84]. Therefore, this can further explain the evidence we found that agreeableness trait is associated with a negative influence on relational aggression. The trait of agreeableness has also been referred to as adaptability or reliability. There are differences in the interpretation of the dimension of agreeableness. The trait of agreeableness is considered reliable whereas Asian people generally support a collectivist culture, emphasizing social harmony and avoiding conflict [84]. Agreeableness represents the obligation to act as a group member and to make sacrifices. This cultural difference can lead to the irrelevance of agreeableness traits against relational aggression among young female adults in Malaysia. Besides that, those with higher levels of neuroticism are thought to be more likely to be aggressive. This individual is considered to have fewer stable emotions. Therefore, people who exhibit many neurotic personality traits are more prone to emotional instability and more prone to conflict with others. Conversely, agreeableness and aggressiveness are consistently negatively correlated [84].

Loneliness shows positively significant prediction towards relational aggression in romantic relationships. This is consistent with the study done by Prinstein et al., [55] which revealed that both relationally aggressive children and youth are more likely to be depressed, lonely, anxious, and socially isolated. However, according to the study done by Povedano et al., [85] found that the relationship between loneliness and relational aggression is significant and positive for boys, but not for girls. The involvement in violent behaviour would not act as a buffer for victimized girls experiencing strong feelings of loneliness, whereas it would be for boys. Lonely people usually have a negative perception of others’ intentions and behaviours in their interpersonal relationships. Along with these findings, lonely people tend to assume that their interpersonal failures stem from unchangeable and undesirable traits in their own personality, and they have a negative interpretation of other people’s intentions and interactions. Individuals who have developed a negative perception of themselves because of loneliness, feeling undesirable and unaccepted by others may resort to relational aggression, a powerful tool in which one uses force in interpersonal relationships to control other people [27].

The results of this study found a positive and significant prediction between avoidant and anxious attachment styles with relational aggression in romantic relationships. It has been established that the quality of communication between parents and children plays a crucial role in the development of a secure attachment. Our findings are in line with previous research that suggests that adolescents who have a positive relationship with their parents and communicate well with them are less likely to engage in aggressive behaviours and engage in risky activities [86]. Moreover, early attachments shape not only an individual’s sense of self and view of the world, but also their social skills, overall well-being, and future relationships. This is supported by the findings of Dervishi et al., [87] who found that adolescents with anxious attachments had higher levels of physical and verbal aggression. Studies have also shown that communication between parents and teens is strongly linked to the emergence of aggressive behaviours, with better communication resulting in a higher sense of security and an active exchange with others throughout life [88–90]. Essentially, individuals who are highly insecure may have difficulties controlling their anger and are more likely to engage in aggressive behaviour.

Previous research has demonstrated that individuals with insecure attachment patterns, particularly the anxious type, are at risk of experiencing negative consequences [91–93]. This can be attributed to a negative self-concept and high levels of rejection anxiety, leading to an over-reaction of excessive anger, and hurt in conflict situations. Research suggests that individuals with anxious attachment style have a history of persistent rejection from their partners and perceive themselves as unworthy of affection [94]. This can result in a perception of partners as untrustworthy and even threatening. It has been found that young adults with anxious attachment style are more prone to experiencing anger, compared to those with a secure or preoccupied attachment style who tend to have more positive expectations of their partners. In other words, those who have a strong sense of insecurity are likely to struggle with controlling their anger, while those with these insecurities are more likely to engage in aggressive behaviour.

Hierarchical regression analysis was carried out and it was found that social support as a moderator showed no significant effect between big five personality, avoidant and anxious attachment style with relational aggression in a romantic relationship except for loneliness subscale. The behaviour’s of loved ones that are in tune with the needs of the individual who is dealing with a stressful situation are referred to as social support [95]. The availability of support in the environment, the emotional response to stressful events, and the assessment of the consequences of these events can all be positively influenced by support from loved ones. Support from loved ones help to decrease the impact of stress by solving the victim’s problems, diminishing the perceived importance of the incident, facilitating the adoption of rational thoughts, and preventing or reducing inappropriate behaviour responses. According to previous research, social support may act as a moderator and buffer the effects of aggression and family functioning [96]. Due to the positive correlation between social support and a person’s family adjustment, social support helps to balance the negative effects of relational aggression on families [97].

This study’s finding is also consistent with the finding by Fortin et al. [98], where the moderating effect of social support is not present in female victims of physical violence. Thompson et al. [99] found that less women who have experienced relational aggression perceive the availability of social support, the more severe the violence they have experienced. The victim may also begin to blame herself more and ask for less support from her loved ones as the violence intensifies due to the bidirectional pattern of violence. Additionally, it seems that continuing in a relationship while having experienced physical abuse may have an impact on how satisfied they are with the assistance they have received [100]. These victims may also require additional forms of support, such as emotional, educational, and material support, even though they are generally happy with the assistance they have received.

Therefore, fewer confidants may have led to less robust social support. As a result, having fewer confidants may have led to social support that was insufficient and did not entirely satisfy the needs of the physical abuse victims. Besides that, social support is thought to be the most important factor that could significantly reduce loneliness [100], and it may be able to predict the trajectory of loneliness [101]. Indeed, numerous studies on the roles played by various forms of social support have found that perceived social support is more useful for predicting people’s mental health and may have a bigger impact on mental health than other forms of social support [102–104].

Both relational aggression and social support are empirically related to levels of loneliness, empirical literature is lacking on the interactive effects of relational aggression and social support on levels of loneliness [53, 105, 106]. Little is devoted in the existing literature to investigating the relationship triad. Ladd and Burgess [52] suggested that social support moderates the association between aggression and adjustment because it balances the dysfunction created by aggression. Family and peer support can act as a buffer in minimizing the negative effects of relational aggression in romantic relationships [107]. Adolescents who receive social support perform better in academic tasks and social interactions than individuals who do not have family and peer support [108]. Consistent with this research, social support, in general, and family support may act as moderating factors for the relationship between levels of loneliness and relational aggression.

Next in this study, it was found that there is no relationship between the role of social support as a moderator in the relationship between attachment style and relational aggression in romantic relationships among young female adults in Malaysia. This is contrary to the results of previous studies that suggest social support act as a moderator and minimizes or increases the effect of relational aggression on parental attachment style because social support is positively related to one’s family adjustment [99] and it has been hypothesized that social support moderates the relationship between relational aggression and parenting style. However, the findings of this current study highlight that social support as a moderator, relational aggression and parenting style are one of the factors that are very influential which affects the functioning of young people based on past studies [104]. The current findings show how social support moderates as an enhancer and buffer in attachment styles and relational aggression.

Results from previous studies differ from the current study due to several factors. Based on attachment style theory by Bowlby (1969), attachment style consists of secure attachment style, anxious attachment style, and avoidance attachment style but in this study only anxious attachment style, and avoidance attachment style alone were used to assess the attachment style of young adults. Avoidant attachment style involves fear of dependence and intimacy interpersonal, excessive need for independence and reluctance to self-disclosure. Anxious attachment styles involve fear of interpersonal rejection or neglect and distress when one’s partner is absent or unresponsive. People with an anxious attachment style always feel insecure about their romantic relationships and fear of abandonment by partner. Those with an avoidant attachment style have a common need to feel loved but not prepared emotionally to be in romantic relationships. Things like this can cause someone to use relational aggression in their romantic relationships such as manipulating partners, threatening partner to end the relationship. In addition, even if that individual has high social support but it does not affect if one is oriented in an avoidant attachment style and anxious attachment style.

Besides that, the findings of this study are consistent with a recent study by Egan and Bull [107] who found that there is no effect of social support as a moderator in the relationship between personality traits and relational aggression in romantic relationships. This is different from the perception based on personality theory developed by Goldberg [109] stating that social support is significantly associated with personality characteristics, especially extraversion, agreeableness, or emotional stability [107]. In general, from childhood to late adulthood, the relationships maintained by individuals with other people are related to individual differences in personality characteristics [110]. Personality traits that define interaction style can predict social interaction, available social support, and its perception. However, a supportive social context may also predict personality traits by providing individuals with opportunities to develop social skills, maintain social relationships, and foster prosocial behaviour. If personal experiences, roles, and social relationships can influence a person’s personality traits, social support is not only a proxy for the quality of social relationships but also a resource that can help to face the social challenges faced in middle adulthood and can predict personality traits by adapting to social roles expectations and developing social skills. Therefore, the relationship between the big five personalities and perceived social support is not only unidirectional but also reciprocal.

### Limitations

As for limitations, all data used in this study were self-reported. The sensitive nature of some questions may have caused some participants to succumb to the social desirability bias and report. For instance, lower rates of relational aggression than their actual behaviour. Despite this, participants provided anonymous answers, making it less likely that they were prompted to provide biased answers. Furthermore, due to recall issues and inaccurate reporting it’s possible that both estimates of psychosocial factors and relational aggression contain measurement error. Another limitation for this study is the cross-sectional nature of these data, which precludes inferences about causal relationships is another drawback of this study.

Additionally, caution should be used when extrapolating the findings to all female samples since the participants in this study were a homogeneous sample of young female adults. Due to the study’s cross-sectional design, it is also impossible to draw conclusions about the cause-and-effect relationship between social support as a moderator in between psychosocial factors and relational aggression. To address the temporal ordering of people’s levels of social support from family and friends and their participation in relational aggression, longitudinal studies are required. Besides that, young female adults were not questioned regarding the opinions or involvement of friends in relational aggression. According to earlier studies, teenagers who have friends who engage in dating violence run a higher risk of doing so themselves [111]. Moreover, data was collected at one time point, so cause-and-effect conclusions could not be made. Besides that, the difference between the psychosocial factor’s groups couldn’t be identified clearly in relation to relational aggression in romantic relationship as only multiple regression has been conducted. A post hoc test can help in identifying the differences between specific groups and give a more meaningful finding.

### Future studies

Future studies are needed on the impact of multiple placements, including their effects on unstable living situations, sibling attachment, adoption, frequent school changes, and difficulties. For instance, if an individual grew up in a family that shamed or condemned emotional expression or in a home with an abusive parent, this may associate anger with fear, danger, or damaged relationships, which will cause to develop more negative perception of their relationship with their parents and siblings. This study only focuses on female samples. Even though there are differences between the genders, both genders naturally experience anger. Men are thought to be more prone to rage despite evidence that women are more emotionally expressive. In addition, more research on gender disparities is necessary.

The current study suggests that preventive measures need to be taken to stop the symptoms of anger from getting worse. Uncontrollable anger can cause several problems, such as erratic behaviour, assault, abuse, addictions, and legal troubles. In these circumstances, anger impairs decision-making, harms relationships, and has other negative effects. Besides that, to manage anger and deal with triggers without repressing and storing it, as well as to deal without causing emotional harm, it’s crucial to recognize the warning signs of anger. Anger management techniques include breathing exercises under supervision, cognitive behavioural therapy, imagery, problem-solving, and the development of interpersonal and communication skills. Besides that, the findings of this study indicate that aimed at reducing and/or preventing relational aggression among young female adults should consider agreeableness traits (112–113). Young female adults who were less agreeable were likely to experience relational aggression. The findings highlight the need for additional research to pinpoint specific characteristics of the lower level of agreeableness female population that put them at risk for relational aggression in a romantic relationship.

The current study was novel in its examination of social support as a moderator of the association between psychosocial factor and relational aggression in romantic relationships. Future studies will need to test these associations further. Based on the findings from this study, there’s no evidence to support the prediction that social support would moderate this association, but future research with a better measure of social support or using different moderator variable may provide different results. Future research should investigate variables that are not included in this study that are possible predictors of relational aggression in romantic relationships. A post hoc test can be conducted further in identifying the differences between specific groups and give a more meaningful finding.

Relational forms of aggression tend to rise during adolescence (115), in part because more complex cognitive abilities are developed during this period that are necessary for successfully manipulating the relationships of others. We discovered a significant correlation between aggression and social support, which is crucial during adolescence. This research suggests that for some people, attachment style and relational aggression are highly overlapping, and possibly reciprocal. However, for some people, personality traits appear to be differentially linked to relational aggression. These results point to the need for additional research examining the moderating effects of significant correlates as well as a more nuanced strategy for relational forms of aggression during early adulthood’s prevention and intervention. Therefore, efforts to prevent young female adults from engaging in relational aggression should concentrate on all females and not just those who have been identified as perpetrators or victims. All females will be better equipped to spot relational aggression signs and help their friends if they are informed about the warning signs of relational aggression. Early adulthood could be taught about the warning signs of relational aggression through community-wide campaigns and in high school. This study will help to create awareness on the existence of relational aggression, public will be able to tackle this issue at an earlier stage rather than later and individuals will be able to identify the difference between a toxic and a non-toxic relationship.

## Conclusion

In conclusion, many participants in this study reported having violent-free romantic relationships even though there are individuals who reported being the perpetrators of relational aggression. The current study was a first step in determining how psychosocial factors and relational aggression in romantic relationships are related to one another. Findings indicate that social support is also an important factor in understanding females’ relational aggression in romantic relationship. At the same time, results demonstrated that social support from friends and/or family has no significant effect with personality traits and attachment styles with relational aggression. This finding raises questions as to what may provide support to young female adults in relational aggression in romantic relationships. The current study’s greatest strength is the dialogue it has sparked about the importance of social support in romantic relationships between young female adults who is experiencing loneliness. This raised awareness could serve as a starting point for further study as well as the creation of programs and regulations that cater to the requirements of this population. It is necessary to create and carry out programs that encourage healthy dating interactions and inform young adults about dating violence which focuses on relational aggression. The findings also provide evidence for the significance of parental modelling in the development of romantic relationships in young adults. The findings are supported by social learning theory (Bandura, 1971), the concepts of which might be employed in investigating other areas of psychosocial factors on young adults’ relationships in the future.

## Data Availability

The datasets used and/or analysed during the current study are available from the corresponding author on reasonable request.

## Abbreviations

BFI: Big Five Inventory
ECR: Experiences in Close Relationships– II
MSPSS: Multidimensional Scale of Perceived Social Support
MRAV: Measure of Relational Aggression and Victimization
UCLA: UCLA Loneliness Scale
